# Gamma heavy chain disease: a retrospective analysis of 6 cases

**DOI:** 10.1186/s13023-023-02679-5

**Published:** 2023-04-11

**Authors:** Long Chang, Dao-bin Zhou, Xin-xin Cao

**Affiliations:** 1 Department of Hematology, Peking Union Medical College Hospital, Chinese Academy of Medical Sciences & Peking Union Medical College, Beijing, China; 2 State Key Laboratory of Complex Severe and Rare Diseases, Peking Union Medical College Hospital, Beijing, China

## To the editor

Heavy chain disease is a group of clinically rare neoplasms of mature B lymphocytes characterized by the production of an abnormally truncated gamma heavy chain protein that lacks the associated light chains. The disease is classified as α, γ, or µ heavy chain disease depending on the type of heavy chain antigen [1]. Gamma heavy chain disease is clinically rare, and the clinical presentation is highly variable, with immunofixation electrophoresis positivity for heavy-chain type M proteins as the only diagnostic basis [2]. There is no widely accepted consensus on the treatment or prognosis of gamma heavy chain disease. In this study, we retrospectively analyzed six patients with gamma heavy chain disease who attended our hospital from January 1, 2012 to December 31, 2021.

Patients with gamma heavy chain disease, diagnosed between January 1, 2012 and December 31, 2021 at Peking Union Medical College Hospital, Chinese Academy of Medical Sciences, were included in this retrospective study. All patients met the diagnostic criteria for gamma heavy chain disease according to the World Health Organization (WHO) 2016 classification [3]. Patients who had histologically confirmed combined lymphoma were diagnosed by lymph node biopsy. All patients completed clinical data collection and follow-up through review of outpatient and inpatient medical records, and telephone inquiries. The latest follow-up date was September 30, 2022. Clinical characteristics, laboratory tests, and treatment used were collected. Bone marrow involvement was defined as aberrant lymphocytes observed in bone marrow flow cytometry or pathology. Overall survival (OS) was defined as the time from diagnosis to the date of death or last follow-up. Progression-free survival (PFS) was defined as the time from diagnosis to disease progression, death or last follow-up.

Of the 6 patients in this group, one was male and five were female, and the median age at diagnosis was 57 (range, 31–70) years. Three patients presented with lymphadenopathy, and one presented with pancytopenia. The other two patients had positive immunofixation electrophoresis results in routine physical examinations. Lymphadenopathy was present in four patients (66.7%), hepatomegaly was present in three patients (50%), and splenomegaly was present in three patients (50%). One patient had an associated lymphoid neoplasm, two had a lymphoid neoplasm and an autoimmune disorder (autoimmune hemolytic anemia and rheumatoid arthritis), and three had only an autoimmune disorder (rheumatoid arthritis and Sjogren syndrome) (Table 1).

**Table 1:** Clinical characteristics, laboratory tests and underlying diseases of 6 patients with gamma heavy chain disease

Patient	Sex	Age (yr)	Associated autoimmune disease	SPEP (g/L)	Hepatomegaly	Splenomegaly	Lymphadenopathy	Bone marrow involvement	Underlying lymphoid neoplasms at diagnosis	Treatment	Outcome
1	Male	40	AIHA	-	+	+	+	+	Small B-cell lymphoma	CHOP	Died of progressive lymphoma 20 months after diagnosis
2	Female	60	RA	-	-	-	--	-	-	W&W	Stable at 52-month follow-up
3	Female	70	RA	20.2	+	+	+	+	B-cell lymphoma, unclassified	CyBorD	Partial response, relapse with DLBCL transformation; treatment with RCHOP regimen, partial response
4	Female	61	SS	-	-	-	-	-	-	W&W	Stable at 42-month follow-up
5	Female	54	SS	8.3	-	-	+	-		W&W	Progression with DLBCL 19 months after diagnosis; treatment with RCHOP, complete response
6	Female	31	-	9.3	+	+	+	+	T-cell lymphoma, NOS	Methotrexate and dexamethasone	Partial response, lost to follow-up 11 months after diagnosis

Abbreviations: AIHA = autoimmune haemolysis anaemia; RA = rheumatoid arthritis; SS = Sjogren syndrome; SPEP = serum protein electrophoresis; R = rituximab; W&W = watch and wait; CHOP = cyclophosphamide, doxorubicin, vincristine, prednisone; CyBorD = cyclophosphamide, bortezomib, dexamethasone; DLBCL = diffuse large B-cell lymphoma; + =present; - =absent

Gamma heavy chain was documented by immunofixation in the serum of all patients. Urine immunofixation electrophoresis was positive in 4 patients. M protein was detected by serum protein electrophoresis in 3 patients, and M protein quantification ranged from 8.3 to 20.2 g. Anemia was found in three patients (50%), and thrombocytosis was found in two patients (33.3%). The median level of hemoglobulin was 98 (range, 64–136) g/L and the median platelet count was 148 (range, 61–173) ×10^9^/L The median level of lactate dehydrogenase was 199 (range, 121–543) U/L, albumin level was 35.5 (range, 22.0–44.0) g/L, IgG level was 16.6 (range, 10.5–31.9) g/L, IgA level was 0.7 (range, 0.4–1.6) g/L, and IgM level was 0.4 (range, 0.1–0.6) g/L. The results of other liver function tests and kidney function tests were normal.

One patient with small B-cell lymphoma received the cyclophosphamide, epirubicin, vincristine plus prednisone (CHOP) regimen, one patient with T-cell lymphoma received the high dose methotrexate (3 g/m^2^) and dexamethasone regimen, and one patient with B-cell lymphoma received the bortezomib, cyclophosphamide plus dexamethasone (CyBorD) regimen. Three patients without lymphoid neoplasms did not receive any treatment. Two of them had no change in clinical and serologic status at the latest follow-up. The other patient had disease progression with diffuse large B-cell lymphoma (DLBCL). Of the three patients treated, one had a partial response (PR) (gamma heavy chain persistence) according to the CT-scan. One had a PR but relapsed with diffuse large B cell lymphoma transformation. One patient died of disease progression. The median duration of follow-up was 32 months (range 12–52). The median OS was not reached. The median PFS was 19 months (range 11–52)(Figure S1).

According to the WHO 2016 classification, heavy chain disease is a mature B lymphocytic neoplasm and can be categorized as α, γ, and µ heavy chain disease according to the heavy chain antigen. The etiology and pathogenesis of heavy chain disease are not well understood and may be related to structural abnormalities of the constant region (CH1) protein in the immunoglobulin G(IgG) heavy chain structure. However, the amount of heavy chain M protein in the serum and urine of patients is often low, and only three of the six patients in this cohort had positive serum protein electrophoresis. Therefore, immunofixation electrophoresis, which is more sensitive, is required for diagnosis. Some new detection techniques, such as capillary electrophoresis and heavy and light chain assays, can improve the sensitivity of heavy chain detection [4]. It is often associated with autoimmune system disorders, such as rheumatoid arthritis, systemic lupus erythematosus, Sjögren syndrome, and myasthenia gravis (Table S1) [5]. In this study, three patients presented with lymphadenopathy and one with pancytopenia, and the other two patients were asymptomatic at diagnosis. In this study, three of the six patients with gamma heavy chain disease had an associated lymphoid neoplasm at diagnosis And five of six patients had a combined autoimmune disorder (autoimmune hemolytic anemia, rheumatoid arthritis and Sjogren syndrome), a higher proportion than previously reported. Perhaps patients with autoimmune disorders were more likely to be tested by immunofixation as a baseline assessment. In addition, combined pulmonary hypertension, systemic sclerosis, inflammatory myopathy, and mixed connective tissue disease were also reported in several cases [6–9].

There is no standard treatment protocol for gamma heavy chain disease due to the large heterogeneity of clinical presentations, and the timing and treatment options depend on the clinical presentation and type of underlying lymphoplasma neoplasm [10]. Asymptomatic patients can be watchfully waited, while symptomatic patients with low-grade lymphomas can be treated with regimens such as chlorambucil, cyclophosphamide and corticosteroids, and aggressive lymphomas can be treated with cyclophosphamide, vincristine plus prednisone (CVP) and etoposide, prednisone, vincristine, cyclophosphamide plus doxorubicin (EPOCH) and CHOP regimen, which can be combined with rituximab for patients with positive CD20 expression [10–13]. The overall prognosis is highly variable, with a median OS of 7.4 years (1 month ~ > 21 years). Of the 3 patients treated in this study, two had PR one of which relapsed with large B-cell transformation, and one died of progression. One of three patients progressed with DLBCL after “watch and wait”.

In conclusion, gamma heavy chain disease was clinically heterogeneous and often combined with autoimmune diseases and lymphoproliferative neoplasms, which should be carefully screened at diagnosis and followed up for aggressive lymphoma transformation by clinicians.

## Electronic supplementary material

Below is the link to the electronic supplementary material.

Supplementary Material 1

Supplementary Material 2

## Data Availability

The datasets during and/or analysed during the current study available from the corresponding author on reasonable request.
