# Supplementary material for: Gamma heavy chain disease: a retrospective analysis of 6 cases
Source: Orphanet J Rare Dis. 2023 Apr 11;18:77. doi: 10.1186/s13023-023-02679-5 (PMC10091613; doi:10.1186/s13023-023-02679-5)
Supplement: Supplementary file 1 — Supplementary Material 1 [file 13023_2023_2679_MOESM1_ESM.docx]

Table S1 Clinical characteristics, laboratory tests and underlying diseases of 6 patients with gamma heavy chain disease.

| **Patient** | **Sex** | **Age (yr)** | **Associated autoimmune disease** | **SPEP (g/L)** | **Hepatomegaly** | **Splenomegaly** | **Lymphadenopathy** | **Bone marrow involvement** | **Underlying lymphoid neoplasms at diagnosis** | **Treatment** | **Outcome** |
| --- | --- | --- | --- | --- | --- | --- | --- | --- | --- | --- | --- |
| 1 | Male | 40 | AIHA | - | + | + | + | + | Small B-cell lymphoma | CHOP | Died of progressive lymphoma 20 months after diagnosis |
| 2 | Female | 60 | RA | - | - | - | -- | - | - | W&W | Stable at 52-month follow-up |
| 3 | Female | 70 | RA | 20.2 | + | + | + | + | B-cell lymphoma, unclassified | CyBorD | Partial response, relapse with DLBCL transformation; treatment with RCHOP regimen, partial response |
| 4 | Female | 61 | SS | - | - | - | - | - | - | W&W | Stable at 42-month follow-up |
| 5 | Female | 54 | SS | 8.3 | - | - | + | - |  | W&W | Progression with DLBCL 19 months after diagnosis; treatment with RCHOP, complete response |
| 6 | Female | 31 | - | 9.3 | + | + | + | + | T-cell lymphoma, NOS | Methotrexate and dexamethasone | Partial response, lost to follow-up 11 months after diagnosis |

Abbreviations: AIHA=autoimmune haemolysis anaemia; RA=rheumatoid arthritis; SS=Sjogren syndrome; SPEP=[serum protein electrophoresis](http://www.baidu.com/link?url=RqLueZElQNDUkE5Iy-vz0j8Iv19p5CrcJA3dxaPG8H4MNnNGRlP3UlKcQKsSOuVkR9M2jFE-Eg9jVGXTEVzO9_IwGzLF2_orCnkFNaWTOuFiUH9xcfhrzDRDy7lPdgL5); R=rituximab; W&W=watch and wait; CHOP=cyclophosphamide, doxorubicin, vincristine, prednisone; CyBorD=cyclophosphamide, bortezomib, dexamethasone; DLBCL=diffuse large B-cell lymphoma; + =present; - =absent.

Table 2. Pathologic diagnosis of underlying disease in patients with gamma heavy chain disease

| Reference | No. of patients | Underlying lymphoid neoplasm | Associated autoimmune disease |
| --- | --- | --- | --- |
| Wahner-Roedler et al. | 23 | Angioimmunoblastic T-cell lymphoma (2), diffuse large B-cell lymphoma (1), Hodgkin lymphoma (1), chronic lymphocytic leukaemia (2), plasmacytoma (3), amyloid (1), unclassified (8) | EVANS syndrome, livedoid vasculitis, rheumatoid arthritis, Sjögren syndrome, vasculitis |
| Bieliauskas et al. | 13 | Unclassified (8), MALT lymphoma (1), splenic diffuse red pulp small B-cell lymphoma (1), splenic marginal-zone lymphoma (1), lymphoplasmacytic lymphoma (1), chronic NK-cell lymphocytosis (1) | Systemic lupus erythematosus (5), autoimmune thyroiditis (3), rheumatoid arthritis (2) |
| Wahbi et al. | 2 | T-cell large granular lymphocytic leukaemia |  |
| Robier et al. | 1 | Chronic lymphocytic leukaemia |  |
| Van Keer et al. | 2 | MGUS |  |
| Iijima et al. | 1 | T-cell large granular lymphocytic leukaemia |  |
| Danic et al. | 1 | MGUS | Rheumatoid arthritis |
| Shibata et al. | 1 | MGUS | Pulmonary hypertension |

Abbreviations: MGUS=monoclonal gammaglobulinemia of undetermined significance; MALT=mucosa-associated lymphoid tissue
