# Supplementary figures and images for: Gamma heavy chain disease: a retrospective analysis of 6 cases
Source: Orphanet J Rare Dis. 2023 Apr 11;18:77. doi: 10.1186/s13023-023-02679-5 (PMC10091613; doi:10.1186/s13023-023-02679-5)

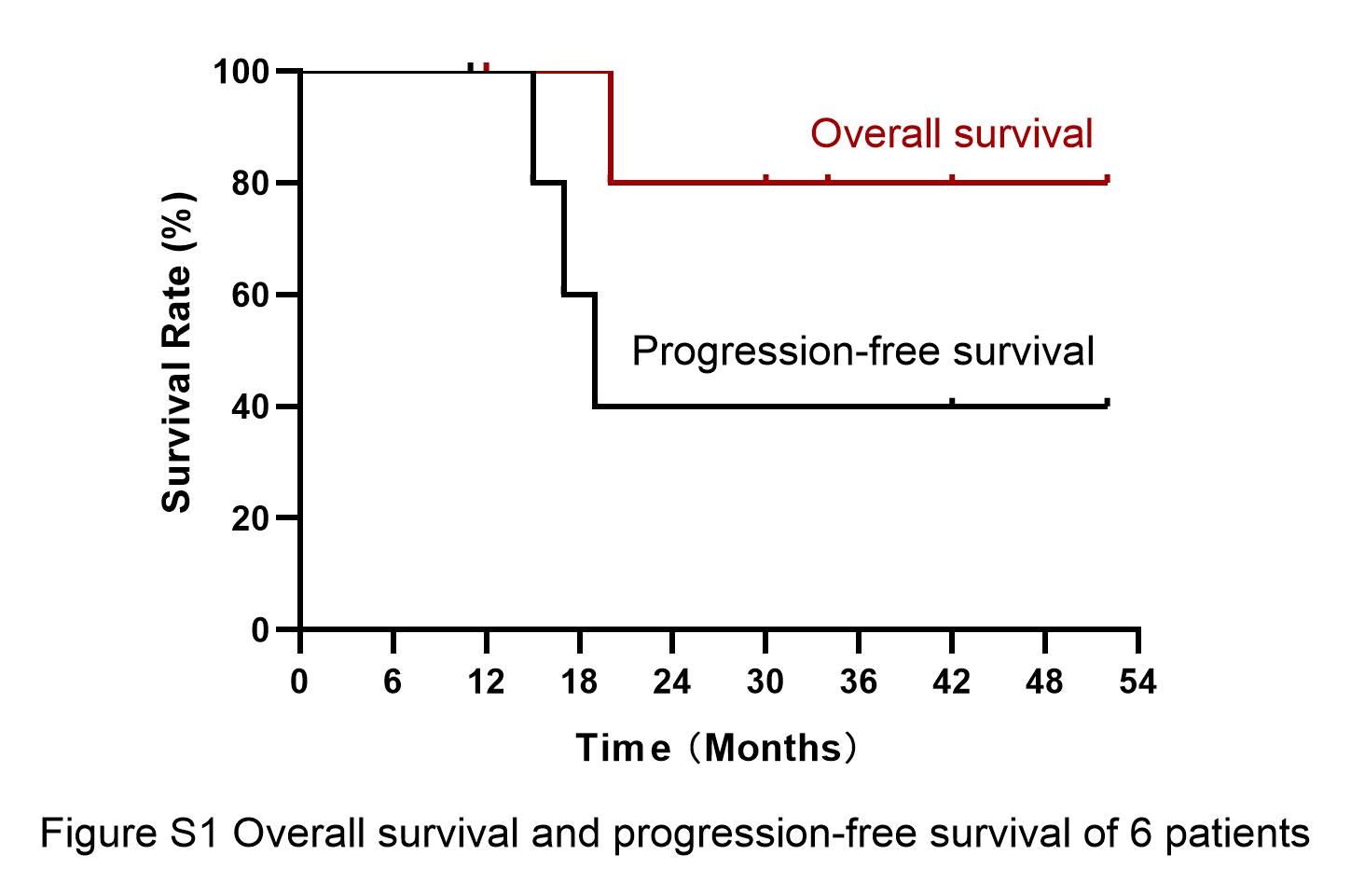

Supplement: Supplementary file 2 — Supplementary Material 2 [file 13023_2023_2679_MOESM2_ESM.jpg]
